# The Effects of Spinal, Inhalation, and Total Intravenous Anesthetic Techniques on Ischemia-Reperfusion Injury in Arthroscopic Knee Surgery

**DOI:** 10.1155/2014/846570

**Published:** 2014-02-20

**Authors:** Müge Koşucu, İlker Coşkun, Ahmet Eroglu, Dilek Kutanis, Ahmet Menteşe, S. Caner Karahan, Emre Baki, Servet Kerimoğlu, Murat Topbas

**Affiliations:** ^1^Department of Anesthesiology, KTU Farabi Hospital, Medical School of Karadeniz Technical University, 61080 Trabzon, Turkey; ^2^Department of Biochemistry, Medical School of Karadeniz Technical University, 61080 Trabzon, Turkey; ^3^Department of Orthopaedics, Medical School of Karadeniz Technical University, 61080 Trabzon, Turkey; ^4^Department of Public Health, Medical School of Karadeniz Technical University, 61080 Trabzon, Turkey

## Abstract

*Purpose*. To compare the effects of different anesthesia techniques on tourniquet-related ischemia-reperfusion by measuring the levels of malondialdehyde (MDA), ischemia-modified albumin (IMA) and neuromuscular side effects. *Methods*. Sixty ASAI-II patients undergoing arthroscopic knee surgery were randomised to three groups. In Group S, intrathecal anesthesia was administered using levobupivacaine. Anesthesia was induced and maintained with sevoflurane in Group I and TIVA with propofol in Group T. Blood samples were obtained before the induction of anesthesia (*t*_1_), 30 min after tourniquet inflation (*t*_2_), immediately before (*t*_3_), and 5 min (*t*_4_), 15 min (*t*_5_), 30 min (*t*_6_), 1 h (*t*_7_), 2 h (*t*_8_), and 6 h (*t*_9_) after tourniquet release. *Results*. MDA and IMA levels increased significantly compared with baseline values in Group S at *t*_2_–*t*_9_ and *t*_2_–*t*_7_. MDA levels in Group T and Group I were significantly lower than those in Group S at *t*_2_–*t*_8_ and *t*_2_–*t*_9_. IMA levels in Group T were significantly lower than those in Group S at *t*_2_–*t*_7_. Postoperatively, a temporary 1/5 loss of strength in dorsiflexion of the ankle was observed in 3 patients in Group S and 1 in Group I. *Conclusions*. TIVA with propofol can make a positive contribution in tourniquet-related ischemia-reperfusion.

## 1. Introduction

A proximal tourniquet is often used in lower limb surgery to provide a bloodless operative field. However, temporary occlusion of arterial blood flow during arthroscopic knee surgery and the subsequent reestablishment of perfusion after deflation of the tourniquet may result in ischemia-reperfusion injury [1–3]. When oxygen is reintroduced into ischemic tissue after tourniquet deflation, massive oxygen radicals are abruptly released into the systemic circulation. Free oxygen radicals can initiate the peroxidation of plasma lipoproteins and polyunsaturated fatty acids in cell membrane macromolecules [1, 4]. Cell injury caused by oxidative stress involves lipids, protein, and DNA, is caused by oxidative stress, and leads to the production of toxic metabolites such as malondialdehyde (MDA) and ischemia-modified albumin (IMA) [1]. MDA is a low molecular weight aldehyde and an intermediate product of lipid peroxidation. Its levels rise as an indicator of lipid peroxidation, and it has often been used as a marker of free radical formation [1–3, 5, 6]. There are many studies [7–11] concerning the rise in IMA levels in acute ischemic conditions, such as myocardial, pulmonary, cerebral, mesenteric, and skeletal muscle ischemia or infarct.

Experimental studies [12–15] have shown that neuromuscular injuries such as skeletal muscle necrosis and axonal degeneration can develop with the use of a tourniquet, especially at a pressure of 200–350 mmHg for 2 h. Tourniquet paralysis of the limb after tourniquet use is a well-recognised complication in the orthopaedic literature. This may be attributed to mechanical damage, soft-tissue oedema, contractures due to cast immobilisation, axonal compression syndrome, and vascular etiology [15, 16]. Clinical studies [16] have demonstrated different degrees of axonal degeneration in the nerves distal to the tourniquet region. The specific injuries reported have included weakness and electromyographic changes in the upper (radial, median, and ulnar) [15] and lower (sciatic, femoral, peroneal, and tibial) limb nerves [14, 16–19]. This means that the decision whether to use a tourniquet has to be made in the light of a profit and loss equation. The measures that might be taken to reduce potential damage in the event of tourniquet use are currently the subject of research.

Earlier studies [1, 3, 4, 6, 7] demonstrated that antioxidants' free radical scavenging activity can restrict lipid peroxidation-related tissue injury. Propofol is one such antioxidant. It is chemically similar to vitamin E, an endogenous antioxidant, and butylated hydroxytoluene, one of the free radical scavengers [1, 20]. It alleviates the effects of stress-inducing hormones, such as adrenaline, noradrenaline, and cortisol [4]. Volatile anesthetics are thought to have a positive effect on free O_2_ radical production in the mitochondrial electron transport chain with a mechanism similar to that of ischemic preconditioning [21]. Some studies [22, 23] suggest that inhalation anesthesia with sevoflurane is associated with less oxidative stress and fewer postoperative complications such as tourniquet-related nerve palsies than spinal anesthesia. However, other publications [24, 25] reported that spinal anesthesia, which is widely used in orthopaedic surgery, suppresses the metabolic response to surgery better than general anesthesia.

Previous studies [1, 2, 5, 11] have shown that both MDA and IMA concentrations rise during lower limb surgery involving a tourniquet; this elevation serves as a marker of ischemia-reperfusion. We hypothesised that an anesthesia technique that reduces MDA and IMA levels, which reflect IRI, might also reduce the side effects of ischemia-reperfusion injury. We thought that decreased ischemia-reperfusion injury might be achieved by a positive contribution of TIVA as a result of using propofol. In this randomised, prospective study, our aim was to evaluate the effect of different anesthetic techniques—TIVA with propofol, inhalation anesthesia with sevoflurane, and spinal anesthesia with levobupivacaine—on tourniquet-related ischemia-reperfusion by determining MDA and IMA levels and side effects in the context of arthroscopic ACL reconstruction surgery.

## 2. Materials and Methods

After obtaining the ethics committee approval and written informed consent from the patients, we studied 60 ASA physical status 1-2 patients, aged between 18 and 55, who were undergoing elective arthroscopic unilateral ACL reconstruction surgery requiring a pneumatic tourniquet. The patients had no metabolic, renal, hepatic, endocrinological, or immunological disturbances and were not using any pharmacological antioxidant agents (including multivitamins, pomegranates, or excessive consumption of tea or coffee) or cigarettes. Patients were allocated randomly by sealed envelope method to one of three groups: spinal anesthesia (Group S, *n* = 20), inhalation anesthesia (Group I, *n* = 20), or total intravenous anesthesia (TIVA) (Group T, *n* = 20).

The patients were not premedicated before surgery. Heart rate (HR), noninvasive arterial blood pressure (BP), peripheral oxygen saturation (SpO_2_), and end-tidal partial pressure of carbon dioxide (ET CO_2_) were monitored in the operating room. Venous access was achieved for anesthesia induction and saline crystalloid solution replacement. A 20 G arterial catheter was inserted on the radial line for blood sampling, and a baseline (*t*_1_) blood sample was taken immediately. In the TIVA group (Group T), anesthesia was induced with propofol 2.5 mg/kg and remifentanil 1–1.5 *μ*g/kg. Anesthesia was maintained with an i.v. infusion of propofol at a rate of 10 mg/kg/h, which was reduced to 8, 6, and 4 mg/kg/h at 10 min intervals and a remifentanil 0.5–1 *μ*g/kg/h i.v. infusion. In the inhalation group (Group I), inhalation anesthesia was induced with sevoflurane mask induction and maintained with sevoflurane at 1.5-fold of the minimum alveolar concentration and 50% nitrous oxide in oxygen. In Groups I and T, i-gel LMA was used without a muscle relaxant, and the lungs were ventilated with 60% air in oxygen (6 mL/kg tidal volume, 10–12 breaths/min, and ETCO_2_ 34–38 mmHg). In the spinal group (Group S), spinal anesthesia was performed with patients in the lateral decubitus position. Lumbar puncture was performed at the L2-3 interspace, using a 27-gauge Quincke bevel spinal needle (Spinocan, B. Braun, Germany); 0.5% plane levobupivacaine 10–12.5 mg was injected over the course of 1 min. During the procedure, a high thigh 9.5 cm circumference by 85 cm length tourniquet was used. The tourniquet was continuously inflated to 300 mmHg on the operative leg and maintained to arrest blood flow. An arthroscopic fluid pump was maintained at the 90 mmHg level throughout the operation. Arterial blood samples were obtained preoperatively from all patients (*t*_1_), 30 min after tourniquet inflation (*t*_2_), immediately before (*t*_3_), and 5 min (*t*_4_), 15 min (*t*_5_), 30 min (*t*_6_), 1 h (*t*_7_), 2 h (*t*_8_), and 6 hours (*t*_9_) after tourniquet release. HR, systolic BP and, SpO_2_ were recorded simultaneously. Mean arterial pressure (MAP) was maintained >70 mmHg, and peripheral oxygen saturation (SpO_2_) was above 95% throughout surgery in all patients. In the spinal group, all patients received 2 L/min of oxygen through a nasal cannula, and ETCO_2_ (infrared spectroscopy) was sampled from one port of the cannula. Target hemodynamic values were defined as an increase or decrease in HR and MAP of more than 25% from baseline. If increases in blood pressure or heart rate exceeded 20%, inhaler gas sevoflurane was planned to be increased at a maximum of 2.5%. In the TIVA group, the propofol infusion rate was planned to be increased gradually. In the spinal anesthesia group, if no results were achieved despite the procedures described above, we sought to bring hemodynamics under control with an additional 1 *μ* gr/kg fentanil. In the event of bradycardia we planned to give 0.01 mg/kg atropine, and in the event of hypotension we planned to first give 500 mL ringer lactate/30 min, and then ephedrine if no result was achieved. All operations were performed by the same surgeon. No intra-articular procedures were performed, and no unusual circumstances or complications were encountered during the operative period. All patients were monitored postoperatively in terms of the development of neurological deficits and motor loss.

The individual examining the blood samples was blinded to the group assignments. For the analysis of MDA and IMA, after plasma had been obtained from the patients, the samples were kept at −80°C until use. Lipid peroxidation in samples was determined as MDA concentrations using the method described by Erturk et al. [1] and Yagi [26]. Briefly, a 2.4 M concentration of N/12 H_2_SO_4_ and 0.3 mL of 10% phosphotungstic acid were added to 0.3 mL of plasma. After incubating at room temperature for 5 min, the mixture was centrifuged at 1600 g for 10 min. Discarded supernatant and sediment were suspended in 4 mL of distilled water. Subsequently, 1 mL of 0.67% thiobarbituric acid was added, and the mixture was heated in boiling water for 60 min. The colour formed was extracted into n-butanol. The mixture was again centrifuged at 1600 g for 10 min. The absorbance of the organic layer was read at 532 nm. Tetramethoxypropane was used as a standard, and MDA levels were calculated as mmol L^−1^. After placing the blood samples in plain tubes containing separation gels, they were allowed to clot for 30 min and centrifuged before separating the serum. The samples were then immediately frozen and stored at −80°C for IMA assays. The results of a reduced cobalt to albumin binding capacity (IMA level) assay were analysed using the rapid and colourimetric method described by Erturk et al. [1] and Bar-Or et al. [27]. Two hundred microliters of patient serum was placed in glass tubes, and 50 mL of 0.1% cobalt chloride (CoCl_2_ *·*6H_2_O; Sigma, St. Louis, MO, USA) in H_2_O was added. After gentle shaking, the solution was incubated for 10 min, to ensure sufficient cobalt albumin binding. Fifty microliters of DTT (1.5 mgml^−1^ of H_2_O, Sigma) was added as a colourising agent, and the reaction was quenched 2 min later by adding 1.0 mL of 0.9% NaCl. A colourimetric control was prepared for preoperative and postoperative serum samples. For the colourimetric control samples, 50 mL of distilled water was substituted for 50 mL of 1.5 mg mL^−1^ DTT. Specimen absorbencies were analysed at 470 nm using a spectrophotometer (Shimadzu UV1601, Auburn, Australia). The colour of the DTT-containing specimens was then compared with that of the colourimetric control tubes. The results were reported as absorbance units (ABSUs).

### 2.1. Statistical Analysis

The sample size was estimated using MDA and IMA levels as the primary endpoint. On the basis of our previous study [1] and assuming an SD of 0.7 mmol L^−1^, 17 patients would be required in each group for an 80% probability of detecting a difference of 0.7 mmol L^−1^ in MDA values at a 5% level of significance.

The Kolmogorov-Smirnov test was used to determine the normality and homogeneity of the data distribution. Parametric data (age, weight, height, tourniquet time, and surgery time) were compared using one-way analysis of variation (ANOVA). Discrete data (sex and ASA) and side effects were compared using the chi-squared test. A repeated measures ANOVA was used for multiple comparison of all 9 repeated measures. Post hoc comparisons were performed using the Tukey test. All of these repeated measurements were compared within groups using the paired *t*-test. Side effects were analysed using the chi-squared test. The results are presented as the means ± SD or number. A *P* value of less than 0.05 was regarded as statistically significant.

## 3. Results

**Figure 1:**
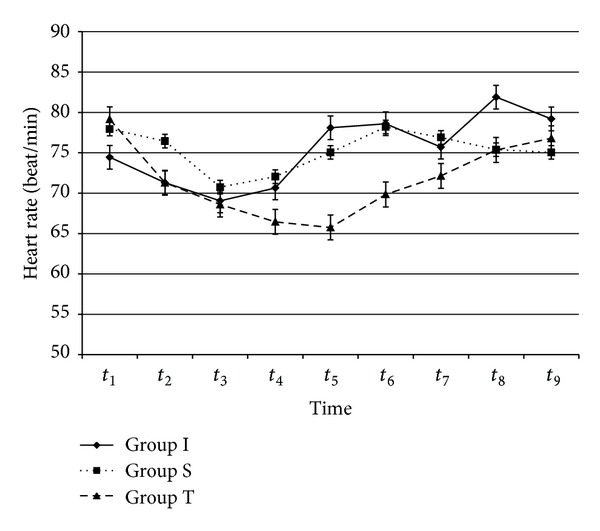
Heart rates values (beat/min). From all patients, preoperatively (*t*_1_), 30 min after tourniquet inflation (*t*_2_), immediately before (*t*_3_), and 5 min (*t*_4_), 15 min (*t*_5_), 30 min (*t*_6_), 1 h (*t*_7_), 2 h (*t*_8_), and 6 hours (*t*_9_) after tourniquet release. *P* > 0.05, for all values.

**Figure 2:**
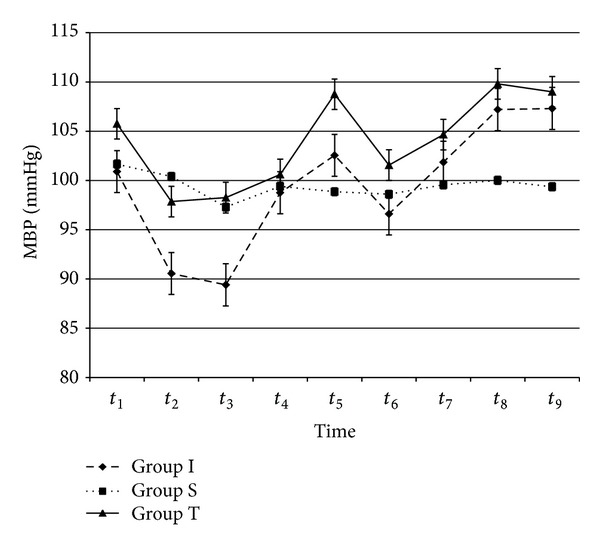
Mean arterial pressure values (mmHg). From all patients, preoperatively (*t*_1_), 30 min after tourniquet inflation (*t*_2_), immediately before (*t*_3_), and 5 min (*t*_4_), 15 min (*t*_5_), 30 min (*t*_6_), 1 h (*t*_7_), 2 h (*t*_8_), and 6 hours (*t*_9_) after tourniquet release. *P* > 0.05, for all values.

As shown in Table 1, there were no significant differences between the groups in terms of age, sex, weight, height, or tourniquet duration. The tourniquet was applied for approximately 66.7 min. Baseline MDA, IMA, and haemodynamic values were not significantly different among the groups. There was no significant difference within or between the groups with respect to HR, MAP, SpO_2_, or ETCO_2_ values (Figures 1 and 2).

Plasma concentrations of MDA increased significantly compared with the baseline values in Group S throughout the period from 30 min after tourniquet inflation (*t*_2_) to 6 h after release (*t*_9_) (*t*_2_–*t*_9_). The plasma concentrations of MDA in Group T were significantly lower than those in Group S, again from 30 min after tourniquet inflation (*t*_2_) to 2 h after release (*t*_8_) (*t*_2_–*t*_8_) (*P* *t*_2_: 0.020, *P* *t*_3_: 0.000, *P* *t*_4_: 0.000, *P* *t*_5_: 0.000, *P* *t*_6_: 0.007, *P* *t*_7_: 0.028, and *P* *t*_8_: 0.010, *P* < 0.05 for all) (Table 2). Plasma MDA concentrations in Group I were significantly lower than those in Group S from 30 min after tourniquet inflation (*t*_2_) to 6 h after release (*t*_9_) (*t*_2_–*t*_9_) (*P* *t*_2_: 0.019, *P* *t*_3_: 0.001, *P* *t*_4_: 0.02, *P* *t*_5_: 0.001, *P* *t*_6_: 0.025, *P* *t*_7_: 0.001, *P* *t*_8_: 0.023, and *P* *t*_9_: 0.022, *P* < 0.05 for all) (Table 2). There were no differences between Group T and Group I.

Plasma IMA concentrations increased in Group S compared with the baseline values from 30 min after tourniquet inflation (*t*_2_) to 1 h after release (*t*_7_) (*t*_2_–*t*_7_). Plasma IMA concentrations in Group T were significantly lower than those in Group S, again from 30 min after inflation (*t*_2_) to 1 h after release (*t*_7_) (*t*_2_–*t*_7_) (*P* *t*_2_: 0.001, *P* *t*_3_: 0.000, *P* *t*_4_ 0.001, *P* *t*_5_: 0.000, *P* *t*_6_: 0.007, and *P* *t*_7_: 0.001, *P* < 0.05 for all) (Table 3). There were no differences between Groups T and I or between Groups I and S.

All patients were evaluated neurologically 3 h after complete recovery from anesthesia. A 1/5 loss of strength in dorsiflexion of the ankle was observed in 3 patients in Group S and 1 in Group I. No loss of strength was observed in any patient in Group T. The difference between the groups was analysed using the chi-squared test, and no difference was observed (chi-squared; 3.75, *P* df2, *P*: 0.153). The loss of strength in dorsiflexion resolved within 1 day after surgery in all patients, so no advanced test (EMG) was performed.

## 4. Discussion

We measured ischemia-reperfusion-associated plasma concentration changes caused by tourniquet use in MDA and IMA, which were regarded as ischemia-reperfusion markers in previous studies. From this prospective, randomised clinical study demonstrated that TIVA with propofol as an anesthetic technique may prevent the increase in the plasma concentrations of IMA and MDA as tourniquet-related ischemia-reperfusion markers in arthroscopic knee surgery.

Propofol is chemically similar to vitamin E, which is an endogen antioxidant, and butylated hydroxytoluene, one of the free radical scavengers [11]. Propofol accumulates in biomembranes, and its radical scavenger activity as a result of the release of a hydrogen atom from its hydroxyl group occurs quite rapidly. It protects erythrocytes against oxidative stress [1, 3, 4, 28]. According to one piece of experimental research [1], propofol significantly inhibits neutrophil infiltration and MDA production in the lungs, which are target organs, after ischemia-reperfusion injury. Earlier studies [1, 3–5] confirmed that propofol alleviated tourniquet-induced ischemia-reperfusion injury in humans compared with other anesthetic agents. Studies [1, 4, 28] have used propofol as a small sedation and anesthetic dose and have demonstrated that these protocols attenuate the production of reactive oxygen species as measured by chemiluminescence in tourniquet-induced ischemia-reperfusion injury. Turan et al. [3] showed a decrease in MDA in the spinal and TIVA groups, in which propofol was used in infusion form. The positive result achieved in the spinal group in that study may be due to the use of propofol as a low-dose infusion rather than as a spinal anesthesia. We applied spinal anesthesia without using propofol or any other drug and identified this group as having the highest MDA and IMA levels of our 3 groups. Because Turan used propofol in induction in the general group and halothane in maintenance, both agents may be considered to have an effect on the outcomes. We used sevoflurane with nitrous oxide as an inhaler agent during the induction and maintenance stages in the inhalation group. In our spinal anesthesia group, which effectively represents the control group, we used no systemic anesthetic drugs, and the highest MDA and IMA levels were observed in this group.

Clinical studies [21, 29] suggest that the combined pre- and postischemic administration of potent inhalation agents has cardioprotective effects against ischemia-reperfusion injury. However, there are few studies in the literature on the physiological effect of volatile anesthetics on the skeletal muscle subjected to tourniquet-induced ischemia-reperfusion injury in humans. The effect of various anesthetic management methods on interstitial glycolysis metabolites (lactate, pyruvate, and glucose) in human skeletal muscle subjected to a tourniquet-related ischemia-reperfusion was evaluated for lower limb surgery [23]. Sevoflurane was found to involve less oxidative stress and fewer postoperative complications, such as tourniquet-related nerve palsies, than spinal anesthesia [22, 23, 30]. Inhalation anesthetic agents and propofol have been compared to examine the effect of MDA levels in different types of surgery. In one study [2], halothane and propofol were compared, and MDA levels were lower in the propofol group, although this effect did not achieve statistical significance. Another similar study [5] compared isoflurane and propofol, and the results again suggested that propofol was superior. Other studies have shown that, with the exception of isoflurane, general anesthetics provide protection against IRI at the coronary and cellular levels. All of these studies compared MDA levels and demonstrated that these were affected by general anesthesia, but they did not compare IMA levels. We investigated both MDA and IMA and observed that sevoflurane inhaled anesthesia caused a fall in MDA and IMA, although the decrease in IMA was not statistically significant. In conclusion, both sevoflurane and, to a greater extent, propofol exhibited a positive effect and lowered the rises in MDA and IMA levels. Arnaoutoglou et al. compared TIVA and inhalation anesthesia and found low MDA in the propofol group, describing this as more effective compared to the sevoflurane group [31]. Budić et al. compared TIVA and inhalation groups with a peripheral nerve block group as the control group [32]. Together with MDA, a lipid peroxidation product, they also investigated catalase activity (CAT), a first line defense mechanism antioxidant enzyme. They observed a positive effect on MDA in the TIVA and PSB groups. However, in contrast to our study, they determined high MDA values in the inhaler anesthesia group. Budić et al. used thiopental and alfentanil in induction in the inhalation group. We used only sevoflurane and nitrogen protoxide, included in the induction stage, and no other iv drug was employed. Thiopental and alfentanil employed may have given rise to these differing results.

Several experimental studies have recently been performed on the effect of remifentanil on ischemia-reperfusion injury [33–36]. These studies have reported positive effects on hepatic and intestinal ischemic reperfusion [33, 34, 36]. Remifentanil reduced hepatic apoptosis and protected against mitochondrial swelling and loss of membrane. It also reduced the TNF alpha and intracellular adhesion molecule-1 induced by ischemia-reperfusion [34]. It has also been shown to be capable of lowering superoxide dismutase and MDA levels [33, 34]. Its effects on the myocardium are unclear; however, although pre- and postconditioning targeting do establish an effect, no positive effect was established in the reperfusion and ischemia-reperfusion periods [35]. Clinical studies are needed to determine the probable effect of remifentanil on ischemia-reperfusion injury.

Literatures comparing spinal and general anesthesia show that the use of spinal anesthesia reduces the frequency of complications [25]. Ischemia-reperfusion injury and neuromuscular complications that may be regarded as-associated can primarily be prevented by techniques that are effective with regard to oxidative stress or anaerobic glycolysis metabolites. Tourniquet-associated neuromuscular deficits have been reported in the literature, with tourniquet pressure and duration being emphasised as the main causes. Our mean tourniquet pressures and durations were similar in the three groups, and there was no statistically significant difference among groups. We observed temporary neurological deficits and motor loss as side effects in 3 patients in Group S and 1 in Group I but none in Group T. Our findings may be important for selecting an anesthetic technique, particularly when a tourniquet is to be used for a longer period of time. However, further clinical studies are now needed to clarify these findings.

Our study had some limitations. First, although we investigated MDA and IMA, both of which are regarded as markers in terms of ischemia-reperfusion in the literature, had we also been able to examine antioxidant enzymes, such as glutathione peroxidase (GPx), catalase (CAT), and superoxide dismutase (SOD), which serve as the first defence mechanism, or lactate, creatine kinase, myoglobin, and troponin as necrosis markers, these might have reinforced the results and helped to elucidate the potential mechanisms involved. In addition, the difference in IMA levels between the spinal and inhalation groups was not statistically significant. Further studies including more patients and operations with longer tourniquet applications may produce more significant results. Additionally, we did not monitor pain and analgesic requirements.

## 5. Conclusion

TIVA with propofol can make a positive contribution in preventing ischemia-reperfusion-associated increases in MDA and IMA in tourniquet-related ischemia-reperfusion in arthroscopic knee surgery. Studies with a longer tourniquet period and a larger number of patients are now needed to better evaluate the effect on clinical practice of this positive contribution.

## Figures and Tables

**Table 1:** Patients characteristic data and tourniquet duration.

	Group I (*n* = 20)	Group S (*n* = 20)	Group T (*n* = 20)	*P* value
Age (year)	44.4 ± 12.4	36.4 ± 13.8	38.1 ± 12.2	0.131
Height (cm)	176 ± 11	170 ± 8	174 ± 10	0.368
Weight (kg)	75 ± 9	74 ± 10	77 ± 7	0.401
Sex (M/F)	9/11	8/12	10/10	0.233
ASA (1/2)	12/8	11/9	13/7	0.291
Tourniquet duration (min)	64.1 ± 18.1	68 ± 30.1	68.1 ± 22.7	0.434

Patients characteristic and surgical data are mean ± SD or number of patients: Group I: ınhalation, Group S: spinal, and Group T: total intravenous anesthesia group.

**Table 2:** Changes in plasma MDA levels.

	*t*_1_	*t*_2_	*t*_3_	*t*_4_	*t*_5_	*t*_6_	*t*_7_	*t*_8_	*t*_9_
Group S	1.8 ± 0.13	3.1 ± 0.14	3.27 ± 0.19	2.9 ± 0.15	3.5 ± 0.17	3.25 ± 0.16	3.25 ± 0.16	3.52 ± 0.17	3.46 ± 0.13
Group I	1.74 ± 0.07	1.92 ± 0.11	1.68 ± 0.09	1.73 ± 0.07	1.91 ± 0.14	2.37 ± 0.14	1.9 ± 0.13	1.83 ± 0.08	2.24 ± 0.17
Group T	1.79 ± 0.06	2.23 ± 0.20	1.59 ± 0.05	1.55 ± 0.06	1.65 ± 0.05	1.99 ± 0.07	2.15 ± 0.09	2.21 ± 0.14	2.43 ± 0.13

*P*	0.250	0.009^a,b^	<0.0005^a,b^	<0.0005^a,b^	<0.0005^a,b^	0.008^a,b^	0.004^a,b^	0.001^a,b^	0.018^a^

Plasma levels of malonyldialdehyde (MDA) (mmol/L). From all patients, preoperatively (*t*_1_), 30 min after tourniquet inflation (*t*_2_), immediately before (*t*_3_), and 5 min (*t*_4_), 15 min (*t*_5_), 30 min (*t*_6_), 1 h (*t*_7_), 2 h (*t*_8_), and 6 hours (*t*_9_) after tourniquet release. (*P* < 0.05; for a: Group T versus S, for b: Group I versus S). Group I: inhalation, Group S: spinal, and Group T: total intravenous anesthesia group.

**Table 3:** Changes in plasma IMA levels.

	*t*_1_	*t*_2_	*t*_3_	*t*_4_	*t*_5_	*t*_6_	*t*_7_	*t*_8_	*t*_9_
Group S	0.24 ± 0.04	0.30 ± 0.04	0.28 ± 0.04	0.29 ± 0.05	0.29 ± 0.05	0.28 ± 0.06	0.28 ± 0.04	0.28 ± 0.05	0.26 ± 0.07
Group I	0.22 ± 0.07	0.26 ± 0.05	0.26 ± 0.05	0.26 ± 0.05	0.25 ± 0.05	0.25 ± 0.04	0.26 ± 0.06	0.26 ± 0.05	0.27 ± 0.04
Group T	0.20 ± 0.07	0.22 ± 0.07	0.20 ± 0.07	0.22 ± 0.07	0.22 ± 0.06	0.22 ± 0.06	0.22 ± 0.05	0.25 ± 0.04	0.24 ± 0.05

*P*	0.220	0.001^a^	<0.0005^a^	0.001^a^	0.001^a^	0.010^a^	0.001^a^	0.126	0.251

Plasma levels of ischemia-modified albumin (IMA) (ABSU). From all patients, preoperatively (*t*_1_), 30 min after tourniquet inflation (*t*_2_), immediately before (*t*_3_), and 5 min (*t*_4_), 15 min (*t*_5_), 30 min (*t*_6_), 1 h (*t*_7_), 2 h (*t*_8_), and 6 hours (*t*_9_) after tourniquet release. *P* < 0.05; for a: Group T versus S. Group I: inhalation, Group S: spinal, and Group T: total intravenous anesthesia group.
